# Regional Difference in Myelination in Monocarboxylate Transporter 8 Deficiency: Case Reports and Literature Review of Cases in Japan

**DOI:** 10.3389/fneur.2021.657820

**Published:** 2021-07-15

**Authors:** Hideyuki Iwayama, Tatsushi Tanaka, Kohei Aoyama, Masaharu Moroto, Shinsuke Adachi, Yasuko Fujisawa, Hiroki Matsuura, Kyoko Takano, Haruo Mizuno, Akihisa Okumura

**Affiliations:** ^1^Department of Pediatrics, School of Medicine, Aichi Medical University, Nagakute, Japan; ^2^Department of Pediatrics and Neonatology, Graduate School of Medical Sciences, Nagoya City University, Nagoya, Japan; ^3^Department of Pediatrics, Fukuchiyama City Hospital, Fukuchiyama, Japan; ^4^Adachi Pediatric Clinic, Fukuchiyama, Japan; ^5^Department of Pediatrics, Hamamatsu University School of Medicine, Hamamatsu, Japan; ^6^Department of Pediatrics, Shinshu University School of Medicine, Nagano, Japan; ^7^Center for Medical Genetics, Shinshu University Hospital, Matsumoto, Japan; ^8^Department of Pediatrics, Fujita Health University School of Medicine, Toyoake, Japan

**Keywords:** magnetic resonance imaging, thyroid hormone transporter, white matter, Allan-Herdon-Dudlley syndrome, reginal analysis, delayed myelination, monocarboxylate transporter 8 deficiency

## Abstract

**Background:** Monocarboxylate transporter 8 (MCT8) is a thyroid hormone transmembrane transporter protein. MCT8 deficiency induces severe X-linked psychomotor retardation. Previous reports have documented delayed myelination in the central white matter (WM) in these patients; however, the regional pattern of myelination has not been fully elucidated. Here, we describe the regional evaluation of myelination in four patients with MCT8 deficiency. We also reviewed the myelination status of previously reported Japanese patients with MCT8 deficiency based on magnetic resonance imaging (MRI).

**Case Reports:** Four patients were genetically diagnosed with MCT8 deficiency at the age of 4–9 months. In infancy, MRI signal of myelination was observed mainly in the cerebellar WM, posterior limb of internal capsule, and the optic radiation. There was progression of myelination with increase in age.

**Discussion:** We identified 36 patients with MCT8 deficiency from 25 families reported from Japan. The available MRI images were obtained at the age of <2 years in 13 patients, between 2 and 4 years in six patients, between 4 and 6 years in three patients, and at ≥6 years in eight patients. Cerebellar WM, posterior limb of internal capsule, and optic radiation showed MRI signal of myelination by the age of 2 years, followed by centrum semiovale and corpus callosum by the age of 4 years. Most regions except for deep anterior WM showed MRI signal of myelination at the age of 6 years.

**Conclusion:** The sequential pattern of myelination in patients with MCT8 deficiency was largely similar to that in normal children; however, delayed myelination of the deep anterior WM was a remarkable finding. Further studies are required to characterize the imaging features of patients with MCT8 deficiency.

## Introduction

Monocarboxylate transporter 8 (MCT8) is a thyroid hormone (TH) transmembrane transporter protein encoded by the *MCT8* (SLC16A2) gene located on human chromosome Xq13.2 (1, 2). Males affected by MCT8 deficiency exhibit severe cognitive deficit, spastic or dystonic quadriplegia, and axial hypotonia, known as the Allan–Herndon–Dudley syndrome (AHDS) (3). An estimated prevalence of MCT8 deficiency is 1 in 70,000 men (4). Disease features in large cohorts have been reported, including brain magnetic resonance imaging (MRI) (4, 5). Brain MRI of patients with MCT8 deficiency shows delayed myelination in the central white matter (WM) (4, 6, 7).

MCT8 is crucial for the transport of triiodothyronine (T3) and thyroxine (T4) in several tissues, including the brain (4). MCT8 is highly expressed in neuronal populations of the cerebral and cerebellar cortex, hippocampus, striatum, and hypothalamus (8). MCT8 is also expressed in the choroid plexus and large capillaries, indicating its involvement in TH transport across the blood–brain barrier (BBB) (9) and/or blood–cerebrospinal fluid (CSF) barrier (8). MCT8 protein is present in neurons and astrocytes of the paraventricular and infundibular nuclei at the human blood–hypothalamus border. Therefore, MCT8 is believed to provide T3 to the central nervous system (CNS) (8).

Oligodendrocytes are the myelinating glia in the CNS (6, 10, 11). Oligodendrocyte precursor cells (OPCs) are established during pre- and perinatal development (6). TH receptors are expressed in the oligodendrocytes in the CNS during embryonic and adult lives (10). The differentiation of OPCs is thought to be regulated by T3 (12). T3 transferred via MCT8 is reported to induce OPC differentiation into mature oligodendrocytes and facilitate the formation of myelin extensions (13). Lack of MCT8 at the OPC level induced reduction of intracellular T3 action, blocking the OPC differentiation to mature and myelinating oligodendrocytes (13). Therefore, abnormal myelination in MCT8 deficiency may be caused by the disruption of OPC differentiation.

Myelination starts early, but the big acceleration phase occurs postnatally up to 2 years of age and is dependent on the brain region. During normal brain development, myelination begins from the 2nd trimester of the fetal life (14–16). Myelination of WM in the brain can be evaluated by conventional MRI. By the age of 3 months, normal infants exhibit high signal intensity in the anterior limb of the internal capsule on brain T1-weighted images (T1WI) (15). On T2-weighted images (T2WI), the splenium of the corpus callosum shows low signal intensity by 6 months of age, the genu of the corpus callosum by 8 months of age, and the anterior limb of the internal capsule by 11 months of age (15). With the exception of the subcortical WM, adult appearance of cerebral WM is seen by the age of 18 months (15).

Patients with MCT8 deficiency are known to exhibit delayed myelination (4). Children with MCT8 deficiency aged <5 years usually show MRI signal of severely delayed myelination, which improve gradually with increase in age (17). MCT8 deficiency was found in 6 of 53 families affected by hypomyelinating leukodystrophies of unknown etiology (18). Nevertheless, analysis of the myelination status in patients with MCT8 deficiency has led to conflicting interpretations (6). The patient heterogeneity in WM phenotype was described (5). Different groups have made different interpretations of the same MRI (7, 19). Hypomyelination rather than delayed myelination has been reported in another study (5). In addition, few studies have characterized the specific myelination pattern in different brain regions in these patients (5). Therefore, regional evaluation using a standardized methodology is necessary to determine the status of myelination in patients with MCT8 deficiency.

In the present study, we describe the regional pattern of myelination in four patients with MCT8 deficiency, based on conventional MRI. In addition, we reviewed the previous case reports of Japanese patients with MCT8 deficiency and assessed the myelination status based on MRI findings.

## Subjects and Methods

### Patients' Background

Case 1 was a nine-month-old child with developmental delay and abnormal thyroid function test (TFT). The child was referred to the Aichi Medical University from regional core hospital (Fukuchiyama City Hospital) for genetic analysis due to suspected MCT8 deficiency. Case 2 was diagnosed as MCT8 deficiency at the age of 9 months due to poor head control and abnormal TFT at the University hospital (Nagoya City University). He was referred to the Aichi Medical University for continuing medical care at the age of 9 months. Cases 3 and 4 were referred from University hospitals (Hamamatsu University Hospital and Shinshu University Hospital) to the Aichi Medical University at the age of 2 years and 3 years, respectively, for the evaluation of TFTs in the neonatal period and the myelination status on MRI.

The clinical course and the TFTs of the four patients with MCT8 deficiency are summarized in Table 1. A cousin of Case 3's mother and the half-brother of Case 4's mother had intellectual disability. Missense mutation of the *MCT8* gene was identified in Cases 1 and 4, while a non-sense mutation was identified in Case 2, and a frameshift mutation was identified in Case 3. All patients showed normal thyroid stimulating hormone (TSH), increased free triiodothyronine (FT3), and decreased free thyroxine (FT4) levels.

**Table 1:** Summary of the clinical course, thyroid function tests, and myelination status in brain T1- and T2-weighted MRI images of the four patients with MCT8 deficiency.

**Case**	**FH**	**Mutation**	**At birth**			**At initial presentation**					**Current condition**						
			**GA**	**BW (g)**	**NBS for IEMs**	**Age**	**Initial symptoms**	TSH (mIU/mL)^*a^	FT3 (pg/mL)^*b^	FT4 (ng/dL)^*c^	**Age**	**TSH (mIU/mL)**	**FT3 (pg/mL)**	**FT4 (ng/dL)**	**Speech**	**Walk**	**Gastric tube**
1	Not remarkable	c.661G>A, p.G221R	41 w	3,190	Normal	4 m	Hypotonia, poor head control, spastic paraparesis	4.979	6.88	0.61	3 y	4.286	7.86	0.54	–	–	–
2	Mother and grandmother had CFS. Father had epilepsy	c.733C>T, p.R245X, the mother shared the same mutation	40 w and 1 d	3,041	Normal	9 m	Poor head control, hypotonia, spastic paraparesis with dystonic/athetoic movements	4.95	8.09	0.75	6 y	3.735	5.63	0.62	–	–	–
3	Mother's cousin had intellectual disability	c.985_986insG, p.D329Gfs^*^2, mother shared the same mutation	37 w	2,790	Normal	4 m	Poor intake, failure to thrive, poor head control, hypotonia, and spastic paraparesis with dystonic/athetoic movements	4.2	9.9	0.5	2 y	NA	NA	NA	–	–	+
4	Mother's half-brother had intellectual disability and died at age of 19 years	c.1556C>T, p.S519L	42 w and 3 d	3,622	Normal	4 m	Poor head control, poor intake, failure to thrive, hypotonia of the trunk, and increased deep tendon reflexes in the extremities	2.92	6.29	0.60	3 y	NA	NA	NA	–	–	–
**Case**	**Age at MRI**	**Imaging sequence**	**Cerebellar WM**	**Internal capsule**		**Optic radiation**	**Corpus callosum**		**Occipital WM**		**Midfrontal WM**		**Anterior frontal WM**		**Centrum semiovale**		
				**Posterior limb**	**Anterior limb**		**Genu**	**Splenium**	**Deep**	**Subcortical**	**Deep**	**Subcortical**	**Deep**	**Subcortical**			
1	8 m	T1	High	Partial high	Iso	high	Iso	Iso	High	Partial high	High	High	High	High	High		
		T2	Iso	Partial low	Iso	Low	Low	Low	High	High	High	High	High	High	High		
	3 y 1m	T1	High	High	High	High	High	High	High	High	High	High	High	High	High		
		T2	Low	Low	Iso	Low	Low	Low	High	High	High	High	High	High	Low		
2	1 y 4 m	T1	High	High	*Iso*	High	Iso	Iso	Partial high	Partial high	Partial high	Partial high	Partial high	Partial high	High		
		T2	Low	Low	Iso	Low	Low	Low	High	High	High	High	High	High	High		
	6 y	T1	High	High	High	High	High	High	High	High	High	High	High	High	High		
		T2	Low	Low	Low	Low	Low	Low	Low	Low	Low	Low	Low	Low	Low		
3	5 m	T1	High	High	Low	High	Low	High	Low	Low	Low	Low	Low	Low	High		
		T2	Low	Low	High	Low	High	Low	High	High	High	High	High	High	Low		
4	6 m	T1^*^	NA	NA	NA	NA	NA	NA	NA	NA	NA	NA	NA	NA	NA		
		T2	Low	Low	Iso	Low	High	Low	High	High	High	High	High	High	High		

*FH, family history; CFS, chronic fatigue syndrome; GA, gestational age; BW, birth weight; NBS for IEMs, newborn screening for inborn errors of metabolism;*

*a *normal range: 0.440–4.000 mIU/mL;*

*b *normal range: 2.20–4.10 pg/mL;*

*c *normal range: 0.80–1.90 ng/dL; NA, not available*.

* *T1 weighted image (T1WI) of this patient was not available because routine examination did not include T1WI*.

### Ethical Compliance

This study was approved by the Institutional Review Board Committee at the Aichi Medical University (H2015-H359). Written informed consent was obtained from the parents. All study evaluations and procedures were performed in accordance with the Declaration of Helsinki.

### Description of Mutations in MCT8

MCT8 encodes two potential proteins comprising of 539 and 613 amino acids depending on the use of two alternative translational start sites in exon 1 (1). Since MCT8 mutations have traditionally been numbered based on the long MCT8 protein, we adhered to this system (1, 16).

### Review of the Literature

We also reviewed previously published cases of MCT8 deficiency reported from Japan. Case reports of Japanese patients with MCT8 deficiency were identified in PubMed and Ichushi-Web (Japanese database of medical literature updated by the Japan Medical Abstracts Society) using the following search terms: Allan–Herndon–Dudley syndrome; MCT8; monocarboxylate transporter 8; SLC16A2. Articles written in English or Japanese published between 2003 and 2020, which described patients with a genetically confirmed MCT8 mutation, were retrieved and the phenotypic and genotypic descriptions were analyzed. If a mutation was described based on a short protein, it was converted to a long protein, as described in the previous section (Description of mutations in MCT8).

### Regional Evaluation on MRI

The initial and current MRI of the four cases and all MRI images available from the published literature were re-evaluated in terms of myelination by two pediatric neurologists. It should be noted that all MRI images of our study's cases were available, whereas for the cases reported in the literature, only those published in the paper were available. Sequential MRIs were available for ten patients. First, two pediatricians, AO and HI, who are authors of this study independently interpreted the MRI images of our four cases and those available in the literature. Subsequently, a consensus on the interpretation was reached after discussion. Cerebellar WM, posterior limb of the internal capsule, anterior limb of the internal capsule, optic radiation, genu corpus callosum, splenium corpus callosum, deep and subcortical occipital WM, deep and subcortical midfrontal WM, deep and subcortical anterior frontal matter, and centrum semiovale were selected for regional evaluation. Signal intensity of WM on T1WI was classified into four grades: high, partial high, iso, and low. Signal intensity of WM on T2WI was classified into four grades: high, iso, partial low, and low. The myelinated appearance was defined as WM showing high intensity on T1WI and low intensity on T2WI.

## Results

### Myelination Status in the Brain MRI Studies of Four Cases

**Figure 1:**
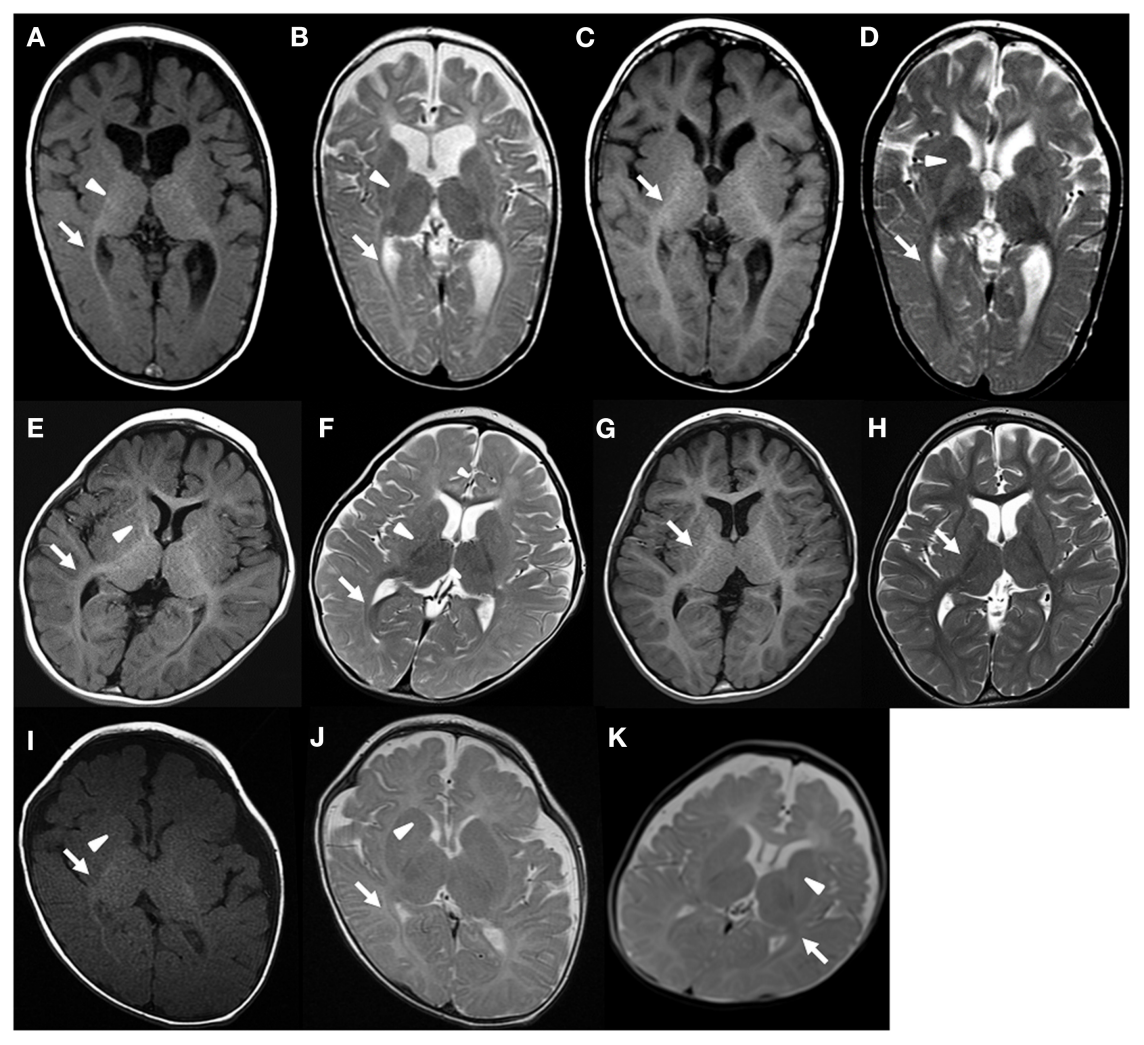
Brain MRI of patient 1 **(A,B)** 8 months, **(C,D)** 3 years and 1 months, patient 2 **(E,F)** 1 year and 4 months, **(G,H)** 6 years, patient 3 **(I,J)** 5 months, and patient 4 **(K)** 6 months. Arrow and arrow head indicates myelination and lack of myelination, respectively. The regional myelination status can be found in Table 1.

In Case 1, optic radiation was myelinated at the age of 8 months (Figures 1A,B). However, other regions were not myelinated (Table 1). At the age of 3 years, MRI signal of myelination was seen in all regions except for the anterior limb of the internal capsule, and the deep and subcortical WM (Figures 1C,D; Table 1).

In Case 2, the cerebellar WM, the posterior limb of the internal capsule, and the optic radiation showed MRI signal of myelination at the age of 16 months (Figures 1E,F). Other regions were not myelinated (Table 1). Brain MRI at the age of 6 years showed myelination of all regions (Figures 1G,H).

In Case 3, MRI signal of myelination was observed in the cerebellar WM, posterior limb of the internal capsule, the optic radiation, splenium corpus, and centrum semiovale at the age of 5 months (Figures 1I,J; Table 1). In Case 4, the cerebellar WM, posterior limb of the internal capsule, and optic radiation showed myelination at the age of 6 months (Figure 1K; Table 1). Follow-up MRI was not available for Cases 3 and 4.

### Review of MRI Images

We identified a total of 36 Japanese patients with MCT8 deficiency from 25 families (Table 2). The median age of patients at the time of reporting was 6.5 years. The age at which MRI and other tests, including TFT, were performed varied in each case. TFT revealed slightly elevated TSH, elevated FT3, and decreased FT4 levels. We reviewed myelination status on T1WI and T2WI. MRI images were available for 30 patients. MRI was performed at the age of <2 years in 13 patients, 2–4 years in 6 patients, 4–6 years in 3 patients, and >6 years in 8 patients (Table 3).

**Table 2:** Summary of the previously reported Japanese patients with MCT8 deficiency.

**Case**	**Author**	**Year**	**Mutation**	**Age**	**TSH**	**FT3**	**FT4**	**Note**
1	Kakinuma et al. (20)	2005	p.S107P	6	3.49	6.82	0.56	
2	Namba et al. (16)	2008	p.Y550Sfs^*^17	3	4	5.5	0.5	
3	Tsurusaki et al. (21)	2011	p.R368X	13	1.2	6.4	1.2	
4	Tsurusaki et al. (21)	2011	p.R368X	8	NA	NA	NA	Sibling to case 3
5	Tsurusaki et al. (21)	2011	p.R368X	Died at 27 y	NA	NA	NA	Cousin to case 3
6	Tsurusaki et al. (21)	2011	p.R368X	Died at 7 m	NA	NA	NA	Cousin to case 3
7	Goto et al. (22)	2013	p.P99Gfs^*^5	4.67	3.782	6.48	0.69	
8	Goto et al. (22)	2013	p.P99Gfs^*^5	NA	NA	NA	NA	Sibling to case 7
9	Yamamoto et al. (23)	2013	Partial deletion	26	2.18	6.3	0.4	
10	Yamamoto et al. (24)	2014	p.P538X	0.58	3.647	7.73	0.52	
11	Yamamoto et al. (24)	2014	p.A224V	0.67	5.93	6.37	0.75	
12	Kobayashi et al. (25)	2014	p.G541C	26	1.3	TT3 2.4^*^	TT4 5.9^*^	
13	Kobayashi et al. (25)	2014	p.G541C	22	1.5	TT3 2.31^*^	TT4 5.7^*^	Cousin to case 12
14	Kobayashi et al. (25)	2014	p.G541C	Died at 32 y	NA	NA	NA	Cousin to case 12
15	Kobayashi et al. (25)	2014	p.G541C	Died at 24 y	NA	NA	NA	Cousin to case 12
16	Morimoto et al. (26)	2014	p.V309L	0.67	6.42	7	0.7	
17	Ono et al. (27)	2016	p.R445S	8	3.1	6.5	0.77	
18	Ono et al. (27)	2016	p.G196E	20	48.5	6.1	0.3	
19	Ono et al. (27)	2016	p.R355Pfs^*^64	21	3.48	5.7	0.6	
20	Shimojima et al. (28)	2016	p.G196V	19	0.8	5.1	0.7	
21	Shimojima et al. (28)	2016	p.G295S	0.5	4.72	10.74	0.59	
22	Yamamoto et al. (29)	2017	p.A252P	1.75	2.23	4.12	1.03	
23	Honda et al. (30)	2017	p.E114X	2	NA	NA	NA	
24	Islam et al. (31)	2019	p.P561X	7	3	5.56	0.607	
25	Islam et al. (31)	2019	p.P561X	0.9	7.67	7.26	0.81	
26	Islam et al. (31)	2019	p.D498N	0.9	5.95	6.78	0.52	
27	Islam et al. (31)	2019	p.G276R	0.5	4.09	7.5	0.7	
28	Islam et al. (31)	2019	p.G276R	1	3.29	9.22	0.64	
29	Islam et al. (31)	2019	p.G276R	1	1.55	6.6	0.65	
30	Islam et al. (31)	2019	p.G401R	2	1.73	7.8	0.5	
31	Islam et al. (31)	2019	p.G312R	1	5.05	8.53	0.65	
32	Islam et al. (31)	2019	p.G312R	1	3.97	9.67	0.66	
33	Iwayama	2020	p.R245X	1.2	4.42	7.41	0.8	This study
34	Iwayama	2020	p.G221R	0.3	4.979	6.88	0.61	This study
35	Iwayama	2020	p.D329Gfs^*^2	0.42	4.2	9.9	0.5	This study
36	Iwayama	2020	p.S519L	0.5	2.92	6.29	0.6	This study
Total			25 families	6.47	5.14	7.05	0.65	

* *Increased total T3 and total T4 within normal range; NA, not available*.

**Table 3:** Myelination status in brain T1- and T2-weighted MRI images in published literature^a*^.

	Cerebellar WM^b*^	**Internal capsule**		**Optic radiation**	**Corpus callosum**		**Occipital WM**		**Midfrontal WM**		**Anterior frontal WM**		**Centrum semiovale**
		**Posterior limb**	**Anterior limb**		**Genu**	**Splenium**	**Deep**	**Subcortical**	**Deep**	**Subcortical**	**Deep**	**Subcortical**	
**High intensity on T1WI**													
<2 years	3/3 (100%)	6/8 (75%)	2/8 (25%)	7/8 (88%)	3/8 (38%)	1/3 (33%)	5/8 (63%)	2/8 (25%)	1/3 (33%)	1/3 (33%)	3/8 (38%)	3/8 (38%)	3/3 (100%)
2–4 years	1/1 (100%)	2/2 (100%)	2/2 (100%)	2/2 (100%)	2/2 (100%)	2/2 (100%)	2/2 (100%)	2/2 (100%)	1/1 (100%)	1/1 (100%)	2/2 (100%)	2/2 (100%)	1/1 (100%)
4–6 years	NA^c*^	1/1 (100%)	1/1 (100%)	1/1 (100%)	1/1 (100%)	NA	1/1 (100%)	1/1 (100%)	NA	NA	1/1 (100%)	1/1 (100%)	NA
>6 years	1/1 (100%)	5/5 (100%)	5/5 (100%)	5/5 (100%)	5/5 (100%)	2/2 (100%)	5/5 (100%)	5/5 (100%)	1/1 (100%)	1/1 (100%)	5/5 (100%)	5/5 (100%)	1/1 (100%)
**Low intensity on T2WI**													
<2 years	3/4 (75%)	11/13 (85%)	1/13 (8%)	11/13 (85%)	9/12 (75%)	4/6 (67%)	1/13 (8%)	0/13 (8%)	0/4 (0%)	0/4 (0%)	0/13 (0%)	0/13 (0%)	1/4 (25%)
2–4 years	1/1 (100%)	3/5 (60%)	1/5 (20%)	2/5 (40%)	5/5 (100%)	2/2 (100%)	0/5 (0%)	0/5 (0%)	0/2 (0%)	0/2 (0%)	0/6 (0%)	0/6 (0%)	1/1 (100%)
4–6 years	NA	3/3 (100%)	2/3 (67%)	2/2 (100%)	3/3 (100%)	2/2 (100%)	2/3 (67%)	1/3 (33%)	NA	NA	0/3 (0%)	1/3 (33%)	NA
>6 years	1/1 (100%)	7/7 (100%)	7/7 (100%)	7/7 (100%)	8/8 (100%)	2/2 (100%)	7/7 (100%)	6/7 (86%)	3/3 (100%)	2/3 (67%)	4/8 (50%)	7/8 (88%)	3/3 (100%)
**Myelinated on MRI**													
<2 years	2/3 (67%)	6/8 (75%)	0/8 (0%)	6/8 (75%)	3/7 (43%)	1/3 (33%)	0/8 (0%)	0/8 (0%)	0/3 (0%)	0/3 (0%)	0/8 (0%)	0/8 (0%)	1/3 (33%)
2–4 years	1/1 (100%)	1/2 (50%)	0/2 (0%)	1/2 (50%)	2/2 (100%)	2/2 (100%)	0/2 (0%)	0/2 (0%)	0/1 (0%)	0/1 (0%)	0/2 (0%)	0/2 (0%)	2/2 100%
4–6 years	NA	1/1 (100%)	1/1 (100%)	1/1 (100%)	1/1 (100%)	NA	1/1 (100%)	0/1 (0%)	NA	NA	0/1 (0%)	0/1 (0%)	NA
>6 years	1/1 (100%)	5/5 (100%)	5/5 (100%)	5/5 (100%)	5/5 (100%)	2/2 (100%)	5/5 (100%)	5/5 (100%)	1/1 (100%)	1/1 (100%)	4/5 (80%)	5/5 (100%)	1/1 (100%)

a* *This list consists of literature data on all patients with MCT8 deficiency on Japanese territory;*

b* *WM, white matter;*

c* *NA, not available*.

On T1WI performed at the age of <2 years, cerebellar WM and centrum semiovale showed high intensity in all patients and posterior limb of internal capsule and optic radiation showed high intensity in most patients. The other regions infrequently exhibited high intensity. In contrast, all regions showed high intensity on T1WI at the age of ≥2 years.

On T2WI performed at the age of <2 years, low intensity was mostly restricted to the cerebellar WM, corpus callosum, posterior limb of the internal capsule, and optic radiation. At the age of 2–4 years, additional low intensity was observed in centrum semiovale. At the age of 4–6 years, low intensity appeared in anterior limb of the internal capsule and deep occipital WM in 2 of 3 patients, and in subcortical occipital, and anterior frontal WM in 1 of 3 patients. At the age of ≥6 years, low intensity was mostly seen in all regions other than the deep anterior WM.

After integration of T1WI and T2WI sequences, cerebellar WM, posterior limb of the internal capsule, and optic radiation were myelinated at the age of <2 years. Then, centrum semiovale and corpus callosum were myelinated at the age of 2–4 years. At 4–6 years of age, anterior limb of the internal capsule and the occipital deep WM had become myelinated. At age >6 years, most regions were found to be myelinated. Considering only T2WI, 50% of patients aged >6 years showed low intensity in the deep anterior WM (Table 3), indicating that myelination was particularly delayed in that area.

## Discussion

We examined regional differences in myelination in four patients with MCT8 deficiency. At the age of <2 years, MRI signal of myelination was observed mainly in the cerebellar WM, posterior limb of the internal capsule, and the optic radiation. There was gradual increase in myelination with further increase in age. On review of MRI images of previously reported Japanese patients, the cerebellar WM, posterior limb of the internal capsule, and optic radiation were myelinated by the age of 2 years, followed by centrum semiovale and corpus callosum by the age of 4 years. Thereafter, most regions with the exception of deep anterior WM showed MRI signal of myelination at the age of 6 years.

### Myelination in Patients With MCT8 Deficiency

Postnatal myelination begins in the cerebellar WM (14–16). In the next few months, posterior limb of the internal capsule, optic radiation, genu corpus callosum, and splenium corpus callosum are myelinated. The anterior limb of the internal capsule is myelinated a little later, by 7–11 months. Myelination progresses from deep and postnatal myelination begins in the cerebellar occipital lobe to the frontal lobe, consecutively. Finally, myelination of the subcortical anterior frontal WM is completed within 2–2.5 years of age.

One of the difficulties in the cases of MCT8 deficiency is that myelination is extremely slow and is therefore often classified as true hypomyelination. In fact, it might take years, but in some cases, myelin restores eventually, against expectations. Autopsy of MCT8-deficient fetus showed a delay in cortical and cerebellar myelination (32). The expression of myelin basic protein (MBP), which is important in the process of myelination, was found to be very low or absent in the MCT8-deficient fetal cerebellum, compared to the control fetus.

Vancamp et al. reviewed data on all literature cases available at that time (6). Abnormal or delayed myelination was reported in 26 out of 31 (84%) patients with MCT8 deficiency aged ≤ 2 years (6). Among patients in the age group of 2–6 years, 63% displayed some form of myelination delay. In our study, delayed myelination was observed in all patients aged <6 years. After the age of 6 years, 67% of patients caught up with the delay and showed full myelination, whereas 33% of patients did not show full myelination but showed partial myelination (6). Remerand et al. reported that out of ten patients with MCT8 deficiency aged >6 years, three had hypomyelination (5). In our study, low intensity on T2WI was not observed in some patients aged <6 years. These data indicated is the presence of patient heterogeneity in myelination after the age of 6 years.

Other papers report subregion differences in myelination (5, 33). Remerand et al. performed regional examination such as enlarged ventricular spaces and hypoplasia of the corpus callosum or cerebellum (5). They also reported a general view of T2 hypersignal on MRI. Matheus et al. reported the myelination status based on the anatomy of WM (33). A comprehensive analysis of myelination status based on WM anatomy will help understand myelination in MCT8 deficiency.

Autopsy study of an 11-year-old boy with MCT8 deficiency showed deficient myelination (32). MRI performed at the age of 6.5 years did not show delayed myelination, although hypomyelination was observed on histopathology at the age of 11 years. Paler MBP staining in the cerebellum was observed in this patient as compared to the control subject. Several other reports have indicated persistence of hypomyelinated areas in some regions in later life (6).

High-resolution MRI detected abnormalities in WM throughout adolescence, suggesting permanent hypomyelination (6). These facts indicate that MRI signal of myelination do not necessarily imply histopathologically complete myelination. Diffusion tensor imaging is reportedly useful in the evaluation of demyelination disease (34) or leukodystrophy (35). More advanced methods for assessment of myelination such as diffusion tensor imaging may help clarify minute abnormalities in myelination in patients with MCT8 deficiency.

### Difference of Myelination According to the Region of the Brain

The myelination status varies in different regions of the brain. In normal brain, MRI signal of myelination is observed in the cerebellar WM, posterior limb of the internal capsule, and the optic radiation by 2 years of age. Myelination of corpus callosum and anterior limb of the internal capsule is observed by the age of 1 year. Subsequently, myelination of WM becomes obvious initially in the deep WM followed by subcortical WM. This study showed the sequential pattern of myelination in patients with MCT8 deficiency was largely similar to that in the normal brain. However, it is remarkable that the MRI signal of myelination was not seen in the deep anterior WM even at the age of 6 years, although subcortical WM showed myelination. T2WI in a 6-year-old patient with MCT8 deficiency showed iso-intensity in the deep anterior WM (33). Conversely, T2WI in a patient with the same age showed low intensity in the deep anterior WM (5).

The primary function of the deep anterior WM is cognition (36). The examples that we have include patients with frontal lobotomy who lose their cognitive capability and become “calm” (37). Furthermore, these symptoms are similar to the patients with MCT8 deficiency. Therefore, we believe that this may explain the good nature of MCT8 deficient children.

The absence of myelination in deep anterior WM has also been proposed in Alexander's disease, which presents as a progressive leukodystrophy (38). The demyelination in Alexander's disease differs from that of MCT8 deficiency in terms of progression and having a remarkably high signal on T1WI. Although the reason for the delayed myelination in the deep anterior WM in patients with MCT8 deficiency is not clear, this finding may be unique to MCT8 deficiency and can be a clue to diagnosis. Further studies are required to validate whether delayed myelination in deep anterior WM may be an imaging feature of MCT8 deficiency.

### Mechanism of Hypomyelination and Recovery of Myelination in MCT8 Deficiency

Oligodendrocytes are the myelinating glia in the CNS (6, 10, 11). OPCs are established during pre- and perinatal development (6). TH receptors are expressed in the oligodendrocytes in the CNS during embryonic and adult lives (10). The differentiation of OPCs is thought to be regulated by T3 (12). T3 transferred via MCT8 was reported to induce differentiation of OPCs into mature oligodendrocytes, and facilitate the formation of myelin extensions (6, 11). Lack of MCT8 at the OPC level induced reduction of intracellular T3 action, blocking the differentiation of OPCs to mature and myelinating oligodendrocytes (6, 11). Therefore, abnormal myelination in MCT8 deficiency may be caused by the disruption of OPC differentiation.

T3 was reported to drive cascade that regulated the timing of OPC differentiation and remyelination of toxic demyelination (39). 3,5-diiodothyropropionic acid (DITPA), which is the analog of TH, can bypass such a deficiency to salvage OPCs and still promote their maturation toward myelinating oligodendrocyte (11). Besides the CNS, hypomyelination occurs in the peripheral nerve system (PNS) due to the lack of T3 in the cell (10). Schwann and satellite glial cells, which are the glial cells of the PNS, transiently express TH receptor only for limited periods of development and regeneration (10). A previous study reports that T3 administration had a positive effect on remyelination in PNS rodent models of inflammatory-demyelinating diseases (40). These data indicate that hypomyelination in MCT8 deficiency might be reversible in the CNS and PNS.

It is difficult to further discuss the mechanisms of spontaneous recovery of myelination, as demonstrated by MRI, and specifically delayed myelination of deep anterior WM as shown in this study. Contrary to the human MCT8 deficiency, a decrease in myelination in a murine MCT8 deficiency model has been reported as permanent (41). In addition, no *in vitro* or animal studies have demonstrated specifically delayed myelination of deep anterior WM. Therefore, the mechanism of spontaneous recovery of myelination and specifically delayed myelination of deep anterior WM remains to be clarified.

### Possible Treatments for MCT8 Deficiency

Physiological or high doses of TH administered postnatally, for most patients even a few years after birth, could not correct hypothyroidism in the brain and psychomotor retardation in MCT8 deficiency (8). Research on several TH analogs, such as DITPA or 3, 3', 5-tri-iodothyroacetic acid (Triac), are still ongoing. DITPA is relatively MCT8 independent for entry into the brain of the MCT8-deficient mouse model (8). Progression in psychomotor development was observed in 2 of 4 patients treated with 22 months of DITPA (42), although their advancement remained at about the same level when expressed as percentage of the chronological age. In two cases with improvement, MRI at 47 months of age showed delayed myelination, which normalized at 62 months of age. It has been reported that motor function in children younger than 4 years treated with Triac was improved (43); however, due to the lack of a control group and the open-label study design, whether the improvement was due to Triac remains unclear. Pre- and post-treatment MRI findings were not reported in the study. Prenatal treatment of intra-amniotic instillation of levothyroxine induced neurodevelopmental improvement and near-normal myelination in the MRI (44). The timing and route of administration, as well as the type of drug, may affect the prognosis. These types of information provide a deeper understanding of myelination in MCT8 deficiency.

### Limitations of This Study

This study had several limitations. First, some of the articles reviewed reported T2WI only. If MRI images were limited to T2WI, fewer MRI images were analyzed. Table 3 shows high intensity at T1WI, low intensity at T2WI, and myelination on MRI. High intensity on T1WI and low intensity on T2WI were considered myelination. However, since imaging changes of myelination occur later in T2 than in T1, even the case of low intensity at T2WI alone was considered as myelination when considering deep anterior WM. Therefore, results in this study may be slightly different from the actual percentage of myelination. Second, due to limited research resources, we evaluated four cases and representative MRI images reported in the literature. The myelination of regions not reported in the literature was evaluated only in our cases. Therefore, results in these regions may not be generalizable. For future study, we plan to centrally evaluate images from Japanese patients with MCT8 deficiency.

## Conclusion

The present study demonstrated regional differences in myelination in patients with MCT8 deficiency based on MRI. The sequential pattern of myelination in different regions of brain in MCT8 deficiency was largely similar to that in normal brain; however, delayed myelination in deep anterior WM was a remarkable feature of MCT8 deficiency. Further studies are required to characterize the imaging features of patients with MCT8 deficiency.

## Data Availability Statement

The original contributions presented in the study are included in the article/supplementary material, further inquiries can be directed to the corresponding author/s.

## Ethics Statement

The studies involving human participants were reviewed and approved by Institutional Review Board Committee at Aichi Medical University. Written informed consent to participate in this study was provided by the participants' legal guardian/next of kin.

## Author Contributions

HI contributed to the study design, interpretation of data, and was a major contributor in writing the manuscript. TT, KA, MM, SA, YF, HMa, and KT contributed to the acquisition and analysis of data from the patients. HMi and AO revised the manuscript critically for important intellectual content. All authors read and approved the final manuscript.

## Conflict of Interest

The authors declare that the research was conducted in the absence of any commercial or financial relationships that could be construed as a potential conflict of interest.
